# The efficacy of alveolar ridge split on implants: a systematic review and meta-analysis

**DOI:** 10.1186/s12903-023-03643-2

**Published:** 2023-11-20

**Authors:** Yuanyou Lin, Guanlin Li, Tingxiang Xu, Xuexiao Zhou, Feng Luo

**Affiliations:** 1 State Key Laboratory of Oral Diseases, West China School of Stomatology, National Clinical Research Center for Oral Diseases, Sichuan University, Chengdu, 610041 China; 2 Department of Prosthodontics, West China School of Stomatology, Sichuan University, No. 14, Section 3, Renmin Nanlu, Chengdu, 610041 China

**Keywords:** Alveolar ridge split, Bone augmentation, Implant survival rate, Gained width, Bone volume, Horizontal ridge deficiency

## Abstract

**Objectives:**

To evaluate the effects of the alveolar ridge split (ARS) technique on gained horizontal width of the alveolar ridge and implant survival rate.

**Materials and methods:**

Electronic searching was performed in six electronic databases (Pubmed, Embase, the Cochrane Central Register of Controlled Trials, Web of Science, China National Knowledge Infrastructure, and SIGLE) from January 1, 2010, to November 1, 2023. Two authors performed study selection, data extraction, and study qualities (ROBINS-I and RoB 2.0) independently. Meta-analysis was performed by Comprehensive meta-analysis 3.0.

**Results:**

24 included studies were observational, and 1 study was a randomized controlled trial (RCT). 14 studies investigated the gained width of the horizontal alveolar ridge, and 17 examined the implants’ survival rate. For assessment of risk of bias, nine studies were high risk of bias and 16 studies were moderate risk of bias. Meta-analysis demonstrated that the pooled gained alveolar ridge width was 3.348 mm (95%CI: 4.163 mm, 2.533 mm), and the implant survival rate was 98.1% (95%CI: 98.9%, 96.9%). Seven studies showed seven different complications including exposure, infection, bad split, dehiscence, fracture, paresthesia and soft tissue retraction.

**Conclusion:**

Recent ARS technique seems to be an effective method of bone augmentation with enough gained width and a high implant survival rate. Further long-term and RCTs research remains needed to enhance the study quality.

**Clinical relevance:**

The ARS technique could generate sufficient bone volume, and implants had a high-level survival rate. Therefore, ARS has been proposed to be a reliable horizontal bone augmentation technique that creates good conditions for the implantation of narrow alveolar crests.

## Introduction

Recently, implant surgery has become integral to dental treatment for patients with dentition defects. Dental implants have become the first choice for more people than ever before. However, not everyone is appropriate for implant surgery. The quantity and quality of alveolar ridges at the implant placement determine an implant’s osseointegration and longevity. Nevertheless, alveolar bone resorption is common after tooth extraction, especially in the maxillae. The buccolingual alveolar ridge dimensions decrease by 3.1 to 5.9 mm 4 to 12 months after extraction [1]. Besides, studies have shown an 11–22% decrease in alveolar bone height and a 29–63% decrease in alveolar bone width in the first 12 months after tooth extraction [2]. In addition, fractures are also a common factor for jaw bone defects. Traffic accidents have been pointed out to be the major incident for fractures especially in the mandible [3]. Therefore, many patients require bone augmentation, especially in horizons.

Several bone augmentation approaches address horizontal bone resorption to achieve successful implantation and long-term results, including bone block grafting, guided bone regeneration (GBR), sinus augmentation and alveolar ridge split (ARS). However, bone block grafting had disadvantages like donor site morbidity and longer treatment time. GBR had the risk of infection due to exposure membrane and collapse of the regeneration membrane [4, 5]. These drawbacks may eventually lead to implant failure. A sinus augmentation was demonstrated to be an effective method for increasing bone height without intraoperative complications even if it was an invasive surgical procedure [6]. Alternatively, the ARS technique may solve some barriers and create proper dimensions for delayed or immediate implantation in both maxillary and mandible. For example, Samieirad et al. successfully performed implant restoration in a severely atrophic maxilla (less than 3 mm) area using bone augmentation with the ARS technique [7]. Besides, Bruschi et al. [8] reported that the success rate of ARS for horizontal bone defects was 98.54% with a minimum 5-year follow-up. These reveal that ARS is a reliable alternative for horizontal bone augmentation.

ARS refers to the surgical procedure of splitting the cortical bone to expand the ridge so implants can be inserted [9]. The method of ridge expansion was first mentioned by Tatum [10] and then modified by Summers [11] with hand osteotomes. The strengths of ARS are shortening treatment duration, decreasing the possibility of infection and morbidity, and being more predictable. ARS seems to be a better choice for narrow alveolar ridge and horizontal bone augmentation. Khoury et al. [12] reported that ARS could achieve an average of 5.2 mm horizontal gained width. Moreover, Jensen et al. [13] found that ARS had a higher implant survival with fewer technical complications than other horizontal bone augmentation methods. However, ARS still had some deficiencies, like fractures, bad splits, and dehiscence.

ARS is the routinely used alveolar bone augmentation technique before dental implant insertion, especially for horizontal ridge deficiency. However, various studies investigating ARS have appeared with multiple clinical outcomes recently. Also, many clinicians are still unfamiliar with the clinical effectiveness of ARS. Therefore, this systematic review aims to evaluate the effectiveness of recent ARS techniques on gained horizontal bone width and implant survival rate until 2010.

## Materials and methods

The systematic review was registered in the International Prospective Register of Systematic Reviews (PROSPERO; Registration number: CRD42022354569).

### Criteria for considering studies for this review

#### Inclusion criteria for eligible studies

Inclusion criteria for included studies were established by the acronym PICOS (Patients, Intervention, Comparison, Outcome, and Study design).

- Patients (P): Completely or partially edentulous patients underwent implant surgery with ARS. There was no gender or age restriction.
- Intervention (I): ARS for bone augmentation in implant surgery.
- Comparison (C): The bone width of the alveolar ridge before and after the ARS and the survival rate of an implant in some period.
- Outcome (O): Primary outcomes: The increased horizontal bone width of the alveolar ridge. Secondary outcomes: The survival rate of implants.
- Study design (S): Randomized controlled trials (RCTs) and nonRCTs (NRCTs).

#### Exclusion criteria

- Nonhuman studies (animals and in vitro studies).
- Studies that the number of patients is less than 10.
- Studies that did not mention the horizontal width of the alveolar ridge and the survival rate of the implant.
- Studies that horizontal width didn’t record either mean or standard deviation.
- Review studies.

### Search strategies and study selection

Two researchers (Mr. Lin and Mr. Li) searched independently in the following databases:

Pubmed, Embase, the Cochrane Central Register of Controlled Trials, Web of Science, China National Knowledge Infrastructure (CNKI), and the grey literature database of SIGLE. The search was from January 1, 2010, to November 1, 2023, without any language restriction. The investigation was conducted by two authors (Mr. Lin and Mr. Li) independently and in duplicate. The specific search strategies are demonstrated in Table 1.

**Table 1:** Search strategy

Step	Strategies
#1 #2 #3 #4 #5	Dental implantation [mesh] OR dental implants [mesh] OR implant* alveolar ridge split* OR lateral ridge split* OR split* alveolar OR ridge expansion OR split crest alveolar width OR gained width OR implant survival rate animal* (#1 AND #2 AND #3) NOT #4

Titles and abstracts were initially screened after duplicates were removed. After assessing full texts, the remaining studies were reevaluated, and final articles were selected. PICOS criteria were followed in the screening process. Two review authors (Mr. Lin and Mr. Li) independently finished the search and studies assessments, and any disagreements were solved by the third review author (Dr. Luo).

Cohen’s unweighted kappa (κ) statistics were used to assess the inter-investigator reliability.

### Data extraction

The general data, including study type, demographic data (age, samples, and sex), intervention, the amounts of implants, and outcomes, were all extracted and recorded independently and duplicated by two authors (Mr. Lin and Mr. Li). Any disagreements were judged by the third review author (Dr. Luo).

Outcomes involved the primary and secondary outcomes–the increased width of the horizontal alveolar ridge after the ARS was regarded as the primary outcome. The survival rate of implants was considered the secondary outcome.

### Statistical analysis

Primary outcome data were put into statistical pooling through random effects models using Comprehensive meta-analysis 3.0. The criteria of data pooling were determined a priori based on comparability of study design, patient type, outcomes measured, treatments, and risk of bias. Mean difference and standard deviation were used for statistical pooling for continuous data. Besides, the event rate was employed for statistical pooling for dichotomus data. For the meta-analyses, both fixed and random-effect models were used to test the reliability of the studies. Then the random-effects model was finally chosen out of conservativeness. In addition, heterogeneity among studies was assessed through the I^2^ statistic, and an I^2^ statistic more significant than 50% was considered substantial heterogeneity.

### Risk of bias assessment

Risk of bias assessment of included studies was performed independently by two authors (Mr. Lin and Mr. Li) using Risk of Bias in Nonrandomized Studies – of Interventions (ROBINS-I) [14] for NRCTs studies and Cochrane Collaborations’ Risk of Bias 2.0 [15] (RoB 2.0) for RCTs studies. In addition, the Begg and Egger test were used to assess the risk of publication bias.

## Results

### Search strategy

**Fig. 1:**
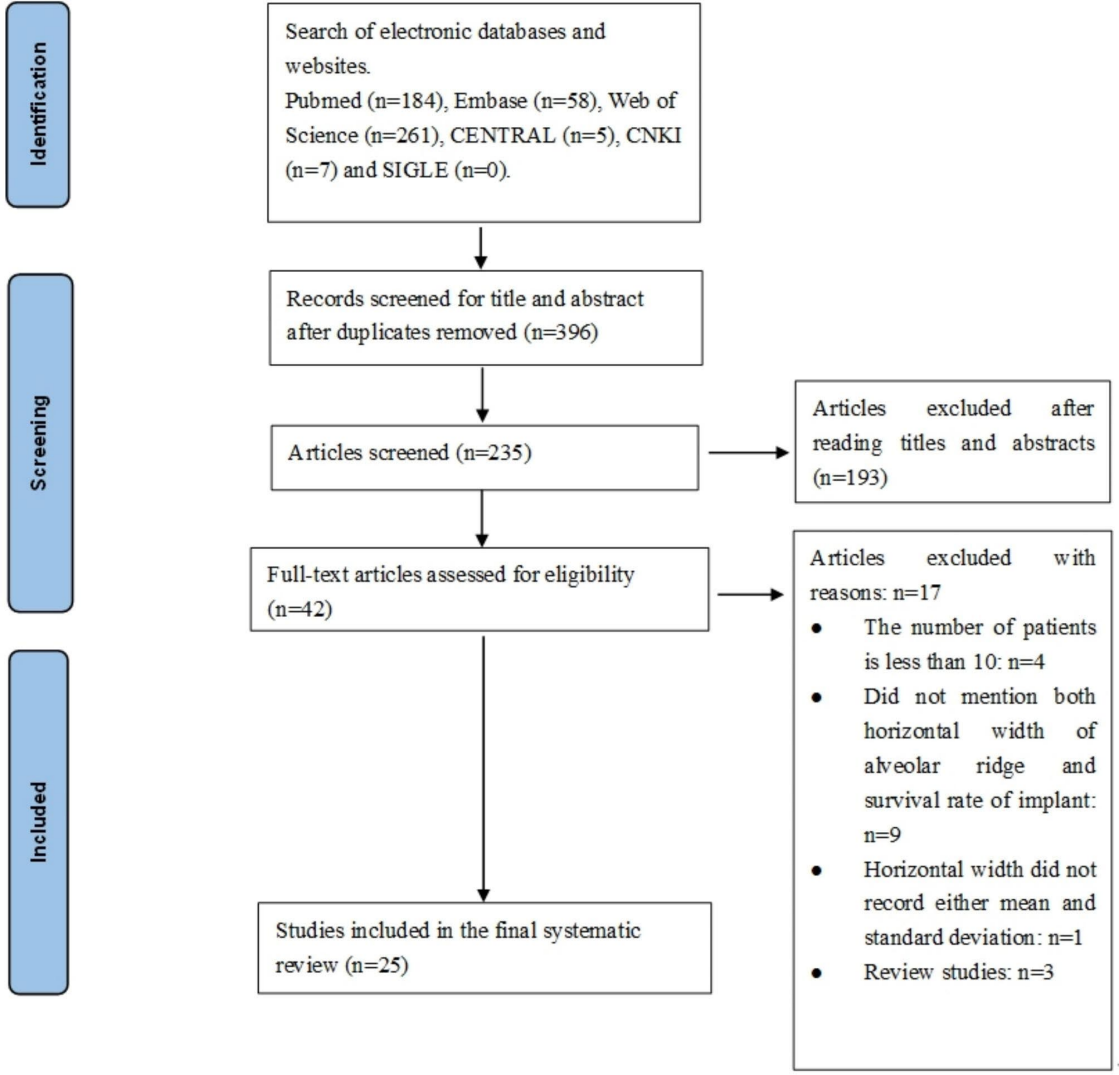
PRISMA flow diagram for processes of studies search and selection

The framework for the literature search and study inclusion is displayed in Fig. 1. The initial electronic search resulted in 235 records upon removal of the duplicates, and no additional studies were identified through a manual search of the gray literature and electronic databases. Of the total studies, 193 articles were excluded after title, abstract, and keyword screening. Upon full-text analysis of the remaining 42 articles, 25 studies [8, 9, 12, 16–36] fulfilled the inclusion criteria and were included in the meta-analysis.

### Description of included studies

The characteristics of all included studies are presented in Table 2. Among 25 studies, 14 studies [9, 12, 16–22, 28–30, 35, 36] reported gained horizontal width of ARS technique, and 17 studies [8, 9, 17, 19–21, 23–27, 30–34, 37] discussed the survival rate of implants. All studies were observation design except 1 RCT [29], 12 [9, 12, 16–18, 21, 22, 30, 32, 33, 35, 36] were prospective, and 12 [8, 19, 20, 23–28, 31, 34, 37] were retrospective studies. 20 studies [9, 12, 16–26, 28, 30, 31, 34–37] used full-thickness flaps to approach the alveolar ridge, the other three studies [8, 27, 32] implemented partial-thickness flaps, and two studies [29, 33] went through flapless surgeries. For the surgery site, only six studies [19, 26, 28, 30, 33, 37] just focused on the mandible, and the other 19 studies [8, 9, 12, 16–18, 20–25, 27, 29, 31, 32, 34–36] involved the maxillae with/without the mandible. 21 included studies [9, 12, 16–21, 23–26, 28–34, 36, 37] reported that bone graft was used in the surgery, including 12 autologous bones and nine xenografts.

**Table 2:** Characteristics of included studies

Reference	Study design	No. of patients	No. of implants	Surgical site	Flap approach	Follow up years	Intervention	Bone graft	Membrane	Gained width	Survival rate
Moro et al. 2017 [16]	Prospective	15 (8 females, 7 males)	32	Maxillae/ Mandible	Full-thickness	6 to 18 months	osteotomy	Autologous bone	resorbable collagen membrane	5.3 ± 1.0	NR
Khoury et al. 2019 [12]	Prospective	142 (90 females, 52 males)	356	Maxillae/ Mandible	Full-thickness	At least 10 years	osteotomy	Autologous bone	NR	5.2± 2.84	NR
Rahpeyma et al. 2013 [17]	Prospective	25	82	Maxillae/ Mandible	Full-thickness	At least 6 months	Osteotomy Immediate implant	Autologous bone	NR	2.0 ± 0.3	100%
Teng et al. 2014 [18]	Prospective	31 (11 females, 20 males)	43	Maxillae	Full-thickness	At least 6 months	osteotomy	Xenograft	Absorbable collagen membrane	2.8± 0.7	NR
Holtzclaw et al. 2010 [19]	Retrospective	13	31	Mandible	Full-thickness	NR	Piezotome osteotomy	Autologous bone	Resorbable collagen membrane	4.03± 0.67	100%
Anitua et al. 2011 [20]	Retrospective	15	37	Maxillae/ Mandible	Full-thickness	11 to 28 months	piezo-surgery Split-crest	Autologous bone	NR	3.35± 0.34	100%
Jamil et al. 2017 [9]	Prospective	23 (18 females, 5 males)	57	Maxillae/ Mandible	Full-thickness	8 to 16 weeks	Piezoelectric osteotomy + immediate implant	Xenograft	Resorbable collagen membrane	4.24± 0.98	100%
Albanese et al. 2019 [21]	Prospective	10	45	Maxillae	Full-thickness	8 to 12 months	Piezoelectric osteotomy + immediate implant	Autologous bone	Double-layer membrane	3.25± 0.94	97.8%
Nguyen et al. 2016 [22]	Prospective	10 (5 females, 5 males)	22	Maxillae	Full-thickness	1 to 3 years	Piezoelectric osteotomy + immediate implant	NR	NR	2.60± 0.40	NR
Manekar et al. 2022 [30]	Prospective	15 (12 females, 3 males)	31	Mandible	Full-thickness	6 to 24 months	crestal osteotomy	Autologous bone	NR	3.2 ± 0.6	100%
Korsakova et al. 2020 [28]	Retrospective	18	39?	Mandible	Full-thickness	6 months	modified two-stage split	Autologous bone	NR	1.6 ± 0.6	NR
Yadav 2022 et al. [36]	Prospective	22 (13 females, 9 males)	22	Maxillae/ Mandible	Full-thickness	6 months	Lateral ridge expansion + immediate implant	Autologous bone	NR	1.98 ± 0.61	NR
Mahmoud et al. 2020 [29]	RCT	557	NR	Maxillae/ Mandible	Full-thickness	6 months	Piezotome osteotomy	synthetic self-hardening biphasic bone graft	NR	4.8 ± 0.6	NR
Wu et al. 2019 [35]	Prospective	36	36	Maxillae	Full- thickness	1-year	U-shape alveolar ridge split	NR	NR	2.56 ± 1.92	NR
Altiparmak et al. 2017 [23]	Retrospective	24 (13 females, 11 males)	43	Maxillae	Full-thickness	38.33 months	Alveolar ridge split	Xenograft	resorbable collagen membrane	NR	100%
Gurler et al. 2017 [24]	Retrospective	17 (12 females, 5 males)	33	Maxillae/ Mandible	Full-thickness	4 to 6 months	Alveolar ridge split	Xenograft	resorbable collagen membrane	NR	93.9%
Garcez-Filho et al. 2014 [25]	Retrospective	21 (12 females, 9 males)	40	Maxillae	Full-thickness	10-year	Alveolar ridge split	Xenograft	NR	NR	97%
Santaga et al. 2016 [32]	Prospective	13 (7female, 6 males.)	33	Maxillae	Partial-thickness	3-year	osteotomy	Xenograft	NR	NR	96.7%
Sohn et al. 2010 [26]	Retrospective	32 (27 females, 5males)	74	Mandible	Full-thickness	3 to 8 months	osteotomy	Xenograft	resorbable collagen membrane	NR	98.8%
Moukrioti et al. 2019 [31]	Retrospective	91 (59 females, 28 males)	173	Maxillae	Full-thickness	3 months	Alveolar ridge split	Autologous bone	NR	NR	100%
Souza et al. 2020 [34]	Retrospective	13	23	Maxillae	Full-thickness	7 to 36 months	Split-crest + immediate implant	Autologous bone	Collagen membrane	NR	100%
Scavia et al. 2019 [33]	Prospective	10	24	Mandible	Flapless	6 months	Piezoelectric osteotomy + immediate implant	Autologous bone	NR	NR	100%
Crespi et al. 2021 [27]	Retrospective	38 (23 females, 15 males)	71	Maxillae/ Mandible	Partial-thickness	5-year	Split crest procedure + immediate implant	NR	NR	NR	98.6%
Bruschi et al. 2017 [7]	Retrospective	71 (39 females, 32 males)	137	Maxillae/ Mandible	Partial-thickness	Minimum 5-year	Split-crest	NR	NR	NR	98.5%
Anitua et al. 2016 [37]	Retrospective	20	31	Mandible	Full-thickness	5-year	Split-crest	Xenograft	fibrin membrane	NR	100%

NR = No report; RCT = Randomized controlled trail;

### Risk of bias assessment

The results of the risk of bias assessment are presented in Table 3. Within 24 non-RCT studies, 8 studies [12, 19, 20, 28, 30, 33, 34, 37] were regarded as having a high risk of bias, and 16 studies [8, 9, 16–18, 21–27, 29, 31, 32, 35, 36] were designated as the overall moderate risk of bias. Of the 1 RCTs, the study [29] had a high risk of bias.

**Table 3:** Risk of bias

Reference	Confounding	Selection bias	Bias in the claasification of interventions	Bias due to deviations from intended interventions	Bias due to missing data	Bias in measurements of outcomes	Bias in selection of the reported result	Overall
Moro et al. 2017 [16]	Mod	Low	Low	Low	Low	Low	Low	Mod
Khoury et al. 2019 [12]	Mod	High	Low	Low	Low	Low	Low	High
Rahpeyma et al. 2013 [17]	Mod	Low	Low	Low	Low	Low	Low	Mod
Teng et al. 2014 [18]	Mod	Low	Low	Low	Low	Low	Low	Mod
Holtzclaw et al. 2010 [19]	Mod	High	Low	Low	Low	Low	Low	High
Anitua et al. 2011 [20]	Mod	High	Low	Low	Low	Low	Low	High
Jamil et al. 2017 [9]	Mod	Low	Low	Low	Low	Low	Low	Mod
Albanese et al. 2019 [21]	Mod	Low	Low	Low	Low	Low	Low	Mod
Nguyen et al. 2016 [22]	Mod	Low	Low	Low	Low	Low	Low	Mod
Manekar et al. 2022 [30]	Mod	High	Low	Low	Low	Low	Low	High
Korsakova et al. 2020 [28]	Mod	High	Low	Low	Low	Low	Low	High
Yadav 2022 et al. [36]	Mod	Low	Low	Low	Low	Low	Low	Mod
Santagata et al. 2015 [32]	Mod	Low	Low	Low	Low	Low	Low	Mod
Wu et al. 2019 [35]	Mod	Low	Low	Low	Low	Low	Low	Mod
Altiparmak et al. 2017 [23]	Mod	Low	Low	Low	Low	Low	Low	Mod
Gurler et al. 2017 [24]	Mod	Low	Low	Low	Low	Low	Low	Mod
Garcez-Filho et al. 2014 [25]	Mod	Low	Low	Low	Low	Low	Low	Mod
Sohn et al. 2010 [26]	Mod	Low	Low	Low	Low	Low	Low	Mod
Moukrioti 2019 [31]	Mod	Low	Low	Low	Low	Low	Low	Mod
Souza et al. 2020 [34]	Mod	High	Low	Low	Low	Low	Low	High
Scavia et al. 2019 [33]	Mod	High	Low	Low	Low	Low	Low	High
Crespi et al. 2021 [27]	Mod	Low	Low	Low	Low	Low	Low	Mod
Bruschi et al. 2017 [7]	Mod	Low	Low	Low	Low	Low	Low	Mod
Anitua et al. 2016 [37]	Mod	High	Low	Low	Low	Low	Low	High

### Meta-analysis

**Fig. 2:**
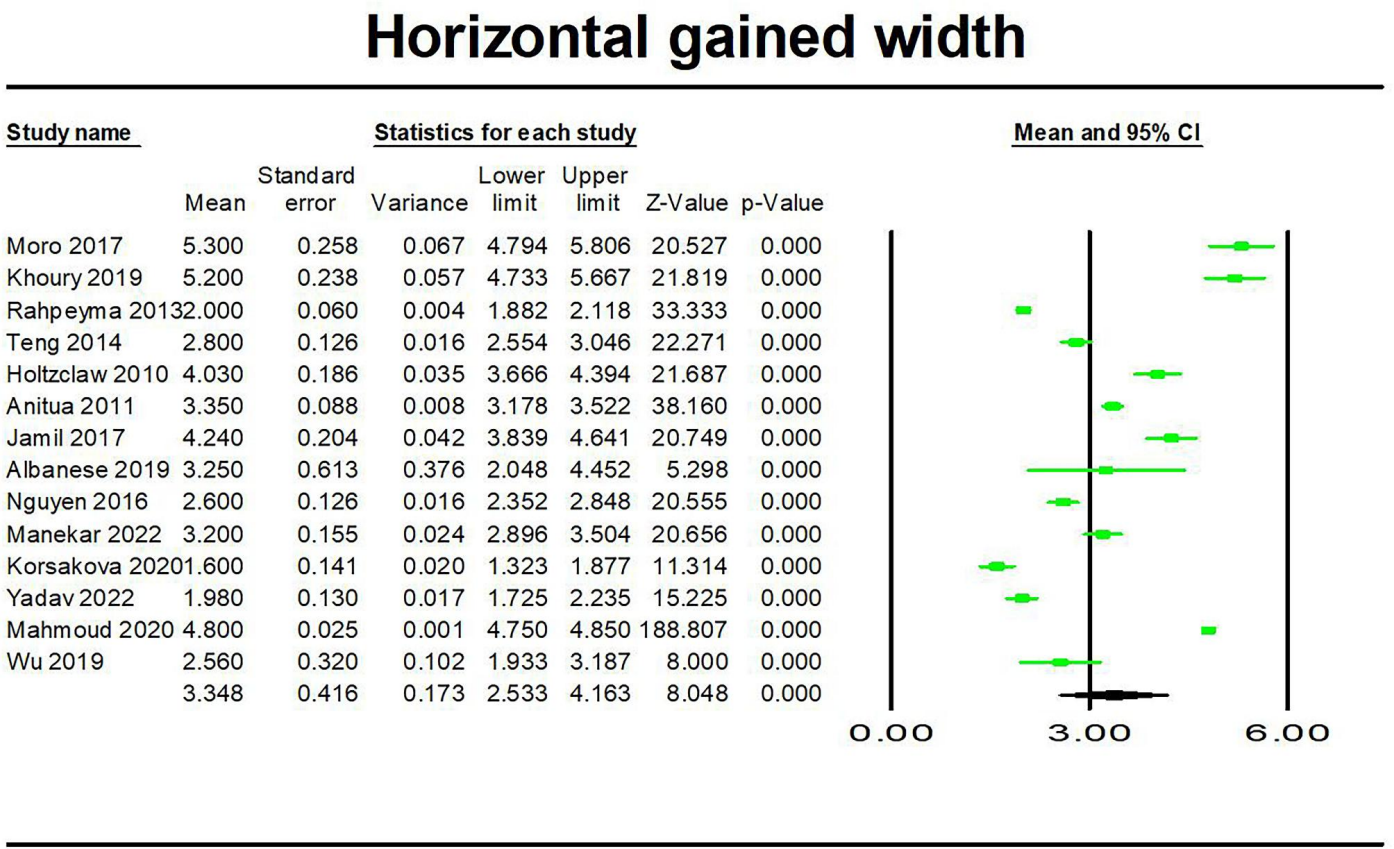
Forest plots of gained width of horizontal alveolar ridge

**Fig. 3:**
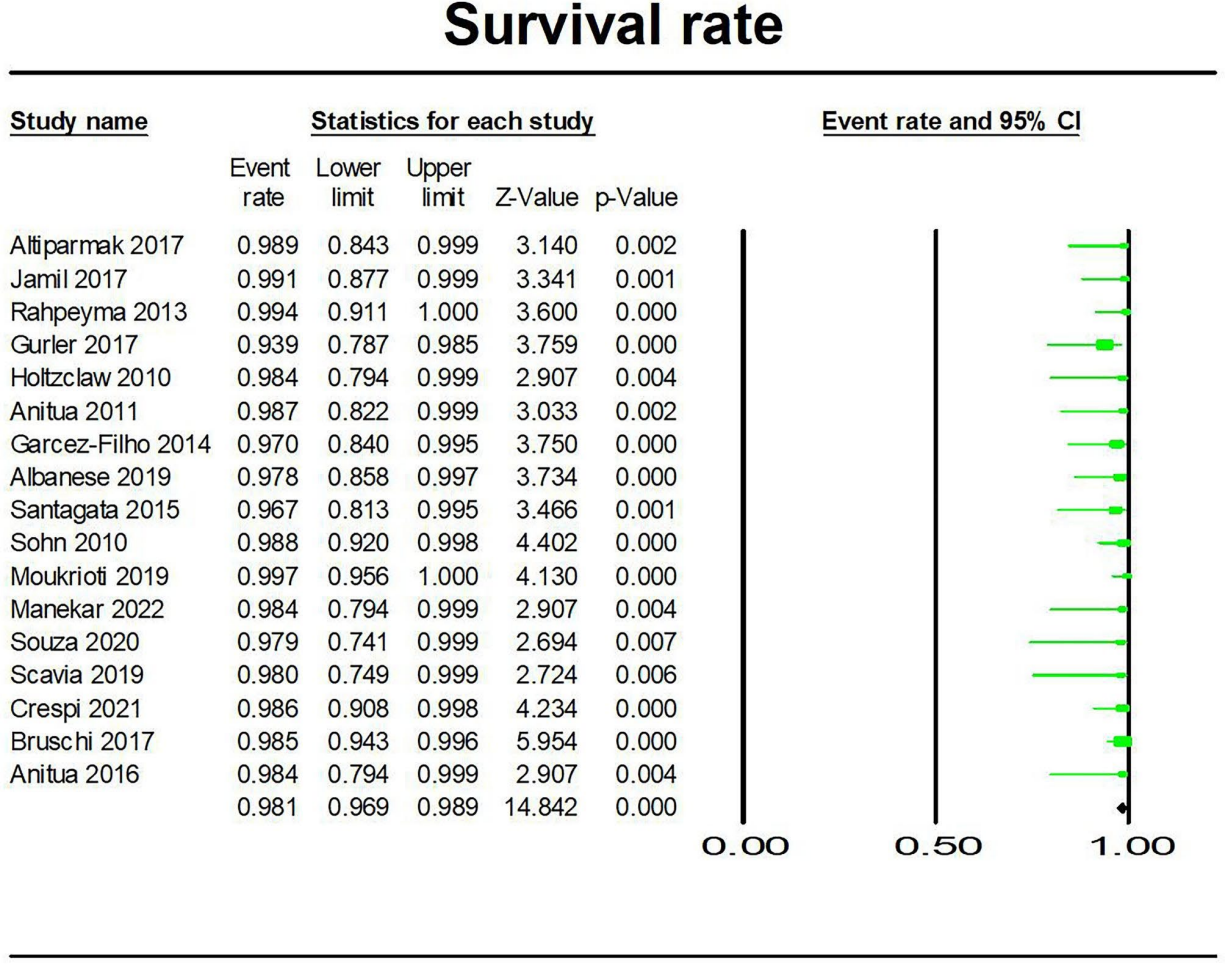
Forest plots of survival rate of implants

We analyzed the gained width of alveolar ridge and implant survival rate of ARS technique. As shown in Figs. 2 and 3, the results were 3.348 mm (95%CI: 4.163 mm, 2.533 mm) for gained horizontal width among 14 studies [9, 12, 16–22, 28–30, 35, 36] and 98.1% (95%CI: 98.9%, 96.9%) for implant survival rate among 17 studies [8, 9, 17, 19–21, 23–27, 30–34, 37].

### Sensitivity analysis

One study removed from the meta-analysis was employed to implement sensitivity analysis. All meta-analysis results demonstrated no significant changes, which represented that the results were robust in this meta-analysis.

### Complications

Overall, seven studies [9, 12, 16, 23, 24, 26, 29] demonstrated seven complications intra or after the surgery including exposure, infection, bad split, dehiscence, fracture, paresthesia and soft tissue retraction (Table 4).

**Table 4:** Description of complications in included studies

Reference	Complications
Moro et al. 2017 [16]	Intraoperative complication: vestibular cortex fracture (1 case). Postoperative complication: membrance exposure (1 case).
Khoury et al. 2019 [12]	heavy bleeding during sinus floor elevation (in 3 sites), a rupture of the sinus membrance up to a diameter of 10 mm (in another 24 sites), small dehuscence (in 6 patients (all smokers)), late bone exposure 4 to 8 weeks postoperatively due to sharp bone borders (in 2 sites(1-3 mm)), infection of the grafted area with abscess and pus (in 1 case), soft tissue retraction on the neighboring tooth at the place of the vertical incision (in 2 cases), early exposure of screws (31 augmented sites(20.13%)).
Rahpeyma et al. 2013 [17]	NR
Teng et al. 2014 [18]	NR
Holtzclaw et al. 2010 [19]	NR
Anitua et al. 2011 [20]	NR
Jamil et al. 2017 [9]	bony dehiscence (at 3 implant sites (5.26%), 1 site in the maxilla and 2 sites in the mandible), buccal plate cracking (only in the mandible at 5 implant sites (8.77%)), transient paresthesia (5 cases:19.23%), early wound dehiscence (4 cases:15.38%), minor soft tissue infections (3 cases:11.54%).
Albanese et al. 2019 [21]	NR
Nguyen et al. 2016 [22]	NR
Manekar et al. 2022 [30]	NR
Korsakova et al. 2020 [28]	NR
Yadav 2022 et al. [36]	Paraesthesia: present in 9.09% (n = 2) subjects
Mahmoud et al. 2020 [29]	irrecoverable surgical site mucosal dehiscence, infection, immediate or delayed spontaneous graft loss, and an indication to remove the graft.
Wu et al. 2019 [35]	NR
Altiparmak et al. 2017 [23]	Minor complications: temporary exposure of the augmented recipient site (2.3%), mild infection (4.7%), a bad spilt (7.1%).
Gurler et al. 2017 [24]	bad split (2 patients), implant failure ( 2 implants failed in 2 patients), wound dehiscence (1 patient).
Garcez-Filho et al. 2014 [25]	NR
Santaga et al. 2016 [32]	NR
Sohn et al. 2010 [26]	NR
Moukrioti et al. 2019 [31]	NR
Souza et al. 2020 [34]	NR
Scavia et al. 2019 [33]	NR
Crespi et al. 2021 [27]	NR
Bruschi et al. 2017 [7]	NR
Anitua et al. 2016 [37]	NR

NR = No report

1. Exposure: Altiparmak et al. [23] found that 2.3% of cases had temporary exposure to the augmented region. Khoury et al. [12] found late bone exposure in 2 sites (1-3 mm) and exposure of screws (20.13%). Moro et al. [16] reported membrane exposure in 1 case.
2. Infection: Altiparmak et al. [23] reported that 4.7% of cases were mild infections. Jamil et al. [9] revealed that 11.54% of cases had minor soft tissue infections. Khoury et al. [12] found that the grafted area with abscess and pus had an infection (1 case). Mahmoud et al. [29] revealed some patients with infections.
3. Bad split: Altiparmak et al. [23] found that 7.1% of cases presented a bad split. Gurler et al. [24] revealed that a bad split was seen in two patients.
4. Dehiscence: Gurler et al. [24] reported that 1 patient had wound dehiscence. Jamil et al. [9] found wound dehiscence in 4 cases (15.38%) and bony dehiscence (1-2 mm from the crest) at 3 implant sites (5.26%). Mahmoud et al. [29] found irrecoverable surgical site mucosal dehiscence in their subjects.
5. Fracture: Moro et al. [16] found a vestibular cortex fracture in 1 case. Sohn et al. [26] found a thin buccal cortical plate fracture in 5 patients.
6. Paresthesia: Jamil et al. [9] found that 19.23 cases had transient paresthesia. Yadav et al. [36] reported 2 patients (9.09%) had paresthesia.
7. Soft tissue retraction: Khoury et al. [12] found soft tissue retraction on the adjacent tooth of the vertical incision in 2 cases.

### Publication bias

The assessment of publication bias is presented in Table 5. For horizontal gained width and survival rate, the Begg and Egger tests demonstrated no evidence of publication bias except for survival rate.

**Table 5:** Publication bias

	Begg Test	Egger Test
Gained width	0.11238	0.06820
Survival rate	0.96409	0.02002

## Discussion

Dental loss, fractures, and pathological processes may cause critical alveolar ridge defects. Patients with severe bone resorption remained a challenge for implantation. Bone augmentation is an important method to ensure the survival and success of implants in patients with defective and atrophic alveolar ridges. ARS technique is considered an effective augmentation method for treating deficient alveolar ridges.

In this study, we systematically reviewed 24 observational and 1 RCT studies [8, 9, 12, 16–37] using the ARS technique to gain horizontal width. The results appealed that ARS could somewhat enhance the horizontal width of alveolar bone. According to the meta-analysis, the width gained from ARS was 3.633 mm within the range of 2.0-5.3 mm. Our results are consistent with the previous studies. For instance, Jensen et al. [38] recommended that the appropriate expansion width of ridge splitting be 3–4 mm. In addition, Crespi et al. [27] indicated a statistically significant increase in the maxillary ridge horizontal width than in the mandible after the split crest technique. It could be explained by the fact that the mean thickness of the buccal wall in the mandible is thinner than half in the maxillary. ARS is a method which splits buccal wall to achieve bone volume. The buccal bone in the maxillary is highly viscoelastic and flexible to minimize the trauma to the bone. Instead, the thinner thickness of mandibular buccal wall which consists of bone cortex leaded to difficult degree of ARS. Furthermore, a case report [39] also presented that the maxillary bone gained more width than the mandible. Maxillary bone which mainly consists of D2, D3 and D4 type bone can be manipulated to appropriate location. However, the bone in the mandible which are mainly D1 and D2 caused obviously difficult for bone manipulation. Manekar et al. [30] revealed that a case (Type 4) with high density of the alveolar bone was not appropriate to be expanded because it caused cervical bone loss. Moreover, a case report [40] presented that the atrophic posterior mandible could be successfully improved by the custom alveolar ridge splitting technique with stable implant placement. Froum et al. [41] also showed the case series that the custom alveolar ridge splitting technique could create intraosseous defect in the atrophic anterior maxilla with successfully implant placement.

On the other hand, the meta-analysis also revealed that the survival rate of implants was 98.1%, within the range of 93.9–100%. It is similar to the rate obtained with standard implant placement procedures. The results indicated that the ARS technique could generate sufficient bone volume, and implants had a high-level survival rate. The implants inserted into expanded ridges using ARS are as successful as those placed into the native, unreconstructed bone. This could be due to sagittal osteotomized ridge gaps undergoing spontaneous ossification following the same procedure as fractures. Thus, ARS (3.63 mm) achieved a lower horizontal bone augmentation width than bone block grafting (4.25 mm) [42]. However, using ARS technology for horizontal bone augmentation can still meet the bone mass requirements for implant placement. Starch-Jensen et al. [13] systematically reviewed the implant treatment outcomes after maxillary alveolar ridge expansion using bone block augmentation versus the ARS technique. The results indicated that the ARS technique could be helpful in horizontal augmentation of the maxillary alveolar bone defect, and the survival rate of prostheses and implants was high. Furthermore, Wu et al. [35] revealed that a new novel U-shape splitting technique (2.56 ± 1.92 mm) could achieve significantly higher gained width than the GBR technique (0.73 ± 1.21 mm). Altiparmak et al. [23] reported that ARS (100%) had a higher survival rate than onlay bone grafting (92%), but there was no significant difference between those two treatment methods. Mahmoud et al. [29] found that there was no significant difference in gained width between autologous bone block grafting (ABBG) and flapless piezotome crest split (FPCS). Still, FPCS has significantly reduced operative time (by > 50%), postoperative pain, and swelling than ABBG. Moreover, more importantly, ARS also have the advantages of immediate implantation and short treatment time. By reducing the healing period, ARS can shorten the length of treatment and provide economic value for patients.

As for complications, this systematic review concluded all included studies with seven complications. However, some complications occurred in an individual study, like paresthesia and soft tissue retraction. The complications in ARS were dehiscence, fracture, exposure, infection, and bad splits. Most complications were well solved by clinicians in the included studies. It also agreed with the previous studies reporting that the common complication for ARS was fracture of the buccal bone [43] and temporary graft exposure [23]. Buccal plate fractures are the most frequent intraoperative complication of ARS, and it has been noted that smaller alveolar bone widths increase fracture risk. As reported, the incidence of fractures increases significantly when the alveolar width is less than 3 mm. Stricker et al. [44] proposed a biomechanical model to mimic the alveolar ridge splitting and a finite element (FE) model to predict maximum lamella displacement to prevent fractures. Samieirad et al. [7] performed a two-step technique to expand the buccal bone and improve the resilience of residual alveolar bone. In this technique, horizontal bone augmentation was performed on the atrophic maxillary anterior ridge by ARS, and then the ridge was expanded, and implants were placed after 6 months. This technique has successfully treated patients with maxillary residual alveolar widths less than 3 mm, and no wound dehiscence or buccal plate fractures were observed. Anitua et al. [37] also demonstrated that delayed implantation with alveolar width narrower than 3 mm could minimize the danger of buccal wall fracture. Furthermore, Goyal and Iyer [45] stated that green stick fracture in the mandible was not controllable owing to the cortical thickness of the bone and the risk of fracture. Compared to the mandible, the thinner cortical plates and softer medullary cancellous bone in the maxilla are more accessible and applicable for ARS. Therefore, many studies recommended two-stage treatment in the mandible. However, Jamil et al. [9] successfully inserted implants into a ridge width of 1 and 1.5 mm and a one-stage approach in the mandible, which was not recommended for the type of surgery. Manekar et al. [30] also smoothly use single-stage alveolar ridge split and expansion (ARSE) to implement immediate implant insertion and reduce the treatment time. Furthermore, the ARS technique with Piezosurgery provided clinicians with immediate implant placement by bone grafting and implant insertion simultaneously. It could essentially reduce the possibility of morbidity, treatment duration, and cost [29].

Even if the present study demonstrated good clinical efficacy, there are still some limitations. Firstly, most human studies did not set a control group and were non-randomized controlled trials. Instead, animal studies have still been conducted but adequately differed from human clinical trials. In addition, short follow-up duration, the limited number of patients, different surgery regions, age, different split technique and clinician experience might be confounding factors to the analysis. Therefore, further studies are supposed to strictly draw up the comparison like (anterior vs. posterior, maxillary vs. mandible, different sex and age), and then cautiously analyze and research the outcomes. Furthermore, factors influencing the results with the ARS technique are also significant to explore.

In this review, the literature search was electronically implemented in six databases. Every method was individually and comprehensively done. We also searched the gray literature to ensure no additional relevant studies were missed, representing an excellent coverage of our topic.

## Conclusion

Through the systematic review and meta-analysis, the recent ARS technique seems to be an effective method for bone augmentation with enough gained width and a high survival rate. Further long-term and RCTs research should be needed to enhance the study quality.

## Data Availability

The datasets used and analysed during the current study are available from the corresponding author on reasonable request.

## Abbreviations

ARS: Alveolar ridge split
GBR: Guided bone regeneration
RCT: Randomized controlled trial
ABBG: Autologous bone block grafting
PFCS: Flapless piezotome crest split
FE: Finite element
ARSE: Alveolar ridge split and expansion
